# Editorial: Bioactive sphingolipids in health and disease

**DOI:** 10.3389/fmolb.2022.1051449

**Published:** 2022-10-11

**Authors:** Mutay Aslan, Yesim Oztas, Liana C. Silva

**Affiliations:** ^1^ Department of Medical Biochemistry, Faculty of Medicine, Akdeniz University, Antalya, Turkey; ^2^ Department of Medical Biochemistry, Faculty of Medicine, Hacettepe University, Ankara, Turkey; ^3^ iMed.ULisboa—Research Institute for Medicines, Faculdade de Farmácia, Universidade de Lisboa, Lisboa, Portugal

**Keywords:** sphingolipid, sphingosine, glycosphingolipid, ceramide, ganglioside

Sphingolipids are structural components of membranes but also important bioactive lipids involved in the regulation of several cellular processes and implicated in many diseases. Sphingolipids consist on a complex aminoalcohol backbone—typically sphingosine—to which a fatty acid molecule is attached yielding ceramide. Phosphocholine linkage to ceramide results in the synthesis of sphingomyelin, while carbohydrate attachment leads to the formation of glycosphingolipids (GSL) (Coant et al., 2017). Though present in minor amounts compared to other sphingolipid species, these complex GSL are nowadays recognized as key players in many pathophysiological processes. This is highlighted in this Research Topic in “Bioactive Sphingolipids in Health and Disease”, where one original research and two review papers are dedicated to these molecules. The review article by Celi et al., focuses on globotriaosylceramide (Gb3), a GSL molecule present in cell membranes of distinct cell types. This work, expands the knowledge of Gb3 function and critically discusses its role in cell membrane structure, cancer, infectious and neurodegenerative diseases. New features for Gb3 from a biological, physiological and immunity point of view with potential for basic research and translation into clinical applications are discussed.

GSL which have one or more sialic acids linked on the carbohydrate chain form gangliosides. Tay-Sachs disease is a rare inherited disorder characterized by GM2 ganglioside accumulation in lysosomes (Fernandes Filho and Shapiro, 2004). Neurological symptoms of the disease are caused by the death of neurons in the brain and spinal cord. Can et al., have analyzed brain lipids in early-onset Tay-Sachs disease mouse model and conclude that understanding of brain-specific lipid alterations may contribute to evaluating the effectiveness of treatments in Tay-Sachs disease mouse model.

Expression patterns of GM3 ganglioside vary during pathogenesis of metabolic syndrome (Tagami et al., 2002). Studies have revealed that GM3 species with differing fatty acid structures act as pro- or anti-inflammatory endogenous toll-like receptor 4 (TLR4) ligands (Inokuchi et al., 2018). Very long-chain fatty acid (VLCFA) and α-hydroxyl VLCFA GM3 variants strongly enhance TLR4 activation. In contrast, long-chain fatty acid (LCFA) and ω-9 unsaturated VLCFA GM3 variants suppress TLR4 activation (Nitta et al., 2019). The review by Inokuchi et al., summarize the findings which demonstrate the pathophysiological significance of GM3 ganglioside with particular emphasis on metabolic syndrome and TLR4 binding.

The less complex sphingolipids and the enzymes involved in their metabolism are also regarded as central molecules in cell function. Dysregulation of early steps of sphingolipid metabolism may cause a broad range of diseases, including metabolic, cancer and neurodegenerative diseases, due to an altered balance between lipid species. There is a considerable amount of studies linking ceramides to the progression and severity of ischemic stroke (Gui et al., 2020). In this Research Topic Ouro et al., have reviewed the involvement of ceramide metabolism in cerebral ischemia. The authors critically discussed the implication of the different enzymes and sphingolipid species on the severity of stroke, and how manipulation of sphingolipid metabolism can exert neuroprotective functions.

The synthesis of ceramide from sphingosine and a fatty acid is catalyzed by ceramide synthases (CerS1-6), which preferentially use a restricted subset of fatty acyl CoAs for N-acylation of sphingosine (Levy and Futerman, 2010). Understanding the function of different CerS can accelerate the process of defining the significance of sphingolipid acyl chain length in cell (patho)physiology. In this Research Topic Sambolín-Escobales et al., have examined the effects of high fat diet (HFD) and short-term unpredictable stress on long-chain ceramides (LCC) in the serum of male and female rats. Though the combined effect of both factors caused a synergistic increase in LCC, no significant changes in depressive-like behaviors were observed. This suggests that increase in serum LCC, as observed in the plasma of patients with major depressive disorder (Brunkhorst-Kanaan et al., 2019), may not be associated with the development of depressive-like behaviors in rodents. Notwithstanding, alterations in ceramide levels contribute to the initiation and progression of inflammatory response and of many diseases. Thus, it is not surprising that sphingolipids may serve as biomarkers that identify dysfunction of physiological processes (Peterson et al., 2018).

In addition to the above-mentioned topics, in this Research Topic also illuminates the role of sphingolipids in tissues and provides new insights into the role of bioactive sphingolipids in health and disease. Finally, we would like to thank all authors for their notable and the referees for their outstanding efforts in supporting this Research Topic.
